# Application of Adipose Stem Cells in 3D Nerve Guidance Conduit Prevents Muscle Atrophy and Improves Distal Muscle Compliance in a Peripheral Nerve Regeneration Model

**DOI:** 10.3390/bioengineering11020184

**Published:** 2024-02-15

**Authors:** Cristian Trâmbițaș, Bogdan Andrei Cordoș, Dorin Constantin Dorobanțu, Cristian Vintilă, Alexandru Petru Ion, Timea Pap, David Camelia, Claudiu Puiac, Emil Marian Arbănași, Claudiu Constantin Ciucanu, Adrian Vasile Mureșan, Eliza Mihaela Arbănași, Eliza Russu

**Affiliations:** 1 Department of Plastic Surgery, George Emil Palade University of Medicine, Pharmacy, Science and Technology of Targu Mures, 540142 Targu Mures, Romania; cristian.trambitas@umfst.ro (C.T.); dorin.dorobantu@umfst.ro (D.C.D.); paptimea96@yahoo.com (T.P.); camelia.david@yahoo.co.uk (D.C.); 2 Clinic of Plastic Surgery, Mures County Emergency Hospital, 540136 Targu Mures, Romania; 3 Veterinary Experimental Base, George Emil Palade University of Medicine, Pharmacy, Science and Technology of Targu Mures, 540139 Targu Mures, Romania; bogdan.cordos@umfst.ro; 4 Regenerative Medicine Laboratory, Centre for Advanced Medical and Pharmaceutical Research (CCAMF), George Emil Palade University of Medicine, Pharmacy, Science and Technology of Targu Mures, 540139 Targu Mures, Romania; emil.arbanasi@umfst.ro; 5 George Emil Palade University of Medicine, Pharmacy, Science and Technology of Targu Mures, 540139 Targu Mures, Romania; 6 Clinic of Anesthesiology and Intensive Care, Mures County Emergency Hospital, 540136 Targu Mures, Romania; claudiu.puiac@umfst.ro; 7 Doctoral School of Medicine and Pharmacy, George Emil Palade University of Medicine, Pharmacy, Science and Technology of Targu Mures, 540139 Targu Mures, Romania; claudio.ciucanu@gmail.com (C.C.C.);; 8 Department of Vascular Surgery, George Emil Palade University of Medicine, Pharmacy, Science and Technology of Targu Mures, 540139 Targu Mures, Romania; adrian.muresan@umfst.ro (A.V.M.); eliza.russu@umfst.ro (E.R.); 9 Clinic of Vascular Surgery, Mures County Emergency Hospital, 540136 Targu Mures, Romania

**Keywords:** peripheral nerve injuries, peripheral nerve regeneration, 3D nerve guidance conduit, uniaxial, mechanical characteristics, stiffness, compliance, muscle atrophy

## Abstract

Background: Peripheral nerve injuries (PNIs) represent a significant clinical problem, and standard approaches to nerve repair have limitations. Recent breakthroughs in 3D printing and stem cell technologies offer a promising solution for nerve regeneration. The main purpose of this study was to examine the biomechanical characteristics in muscle tissue distal to a nerve defect in a murine model of peripheral nerve regeneration from physiological stress to failure. Methods: In this experimental study, we enrolled 18 Wistar rats in which we created a 10 mm sciatic nerve defect. Furthermore, we divided them into three groups as follows: in Group 1, we used 3D nerve guidance conduits (NGCs) and adipose stem cells (ASCs) in seven rats; in Group 2, we used only 3D NGCs for seven rats; and in Group 3, we created only the defect in four rats. We monitored the degree of atrophy at 4, 8, and 12 weeks by measuring the diameter of the tibialis anterior (TA) muscle. At the end of 12 weeks, we took the TA muscle and analyzed it uniaxially at 10% stretch until failure. Results: In the group of animals with 3D NGCs and ASCs, we recorded the lowest degree of atrophy at 4 weeks, 8 weeks, and 12 weeks after nerve reconstruction. At 10% stretch, the control group had the highest Cauchy stress values compared to the 3D NGC group (0.164 MPa vs. 0.141 MPa, *p* = 0.007) and the 3D NGC + ASC group (0.164 MPa vs. 0.123 MPa, *p* = 0.007). In addition, we found that the control group (1.763 MPa) had the highest TA muscle stiffness, followed by the 3D NGC group (1.412 MPa), with the best muscle elasticity showing in the group in which we used 3D NGC + ASC (1.147 MPa). At failure, TA muscle samples from the 3D NGC + ASC group demonstrated better compliance and a higher degree of elasticity compared to the other two groups (*p* = 0.002 and *p* = 0.008). Conclusions: Our study demonstrates that the combination of 3D NGC and ASC increases the process of nerve regeneration and significantly improves the compliance and mechanical characteristics of muscle tissue distal to the injury site in a PNI murine model.

## 1. Introduction

Peripheral nerve injuries (PNIs) represent a significant clinical problem that can lead to loss of function and sensation [1,2,3]. Traditional approaches to nerve repair, such as autografts and allografts, have limitations, such as donor site morbidity and the limited availability of donor nerves [3,4,5]. Recent advances in 3D printing and stem cell technology offer a promising solution for nerve regeneration by developing 3D-printed scaffolds that can support the growth and differentiation of stem cells. By incorporating stem cells into 3D-printed scaffolds, it is possible to promote nerve regeneration and improve the functional outcomes of patients with PNI [6,7,8,9].

Several studies have shown the potential of 3D printing and stem cells in nerve regeneration. Researchers have successfully demonstrated the use of 3D-printed scaffolds to guide the growth of axons and support Schwann cells in the regeneration of nerves. Stem cells have also been used to enhance nerve regeneration by promoting the differentiation of Schwann cells and producing neurotrophic factors [10,11,12]. Despite promising results, there are still several challenges to be addressed in the field of 3D printing and stem cells for nerve regeneration. These include optimizing the scaffold design and materials, improving cell viability and differentiation, and developing effective methods for delivering stem cells to the site of injury [9,13].

Recently, the efficiency and feasibility of biodegradable and biocompatible polymers have been analyzed in nerve regeneration [14,15,16,17]. Amongst the various polymers examined, Polylactide-co-glycolide acid (PLGA) has shown the most promising outcomes [14,15,16]. However, the utilization of PLGA grafts did not significantly promote nerve regeneration, according to an in vivo study conducted by Pozzobon et al. [17].

Another crucial aspect of post-operative and post-traumatic recovery is the degree of muscle atrophy and the compliance of the muscle tissue [18,19,20]. According to Choe et al. [21], the ipsilateral limb had a more pronounced degree of muscle atrophy compared to the contralateral limb in a rat neuropathic pain model.

The main purpose of this study is to analyze the biomechanical characteristics, from physiological stress to failure, in muscle tissue distal to a nerve defect in a murine model of peripheral nerve regeneration, involving both nerve reconstruction using a 3D nerve guide conduit as well as the additional application of adipose-derived stem cells. Furthermore, this study aims to assess the extent of muscle atrophy based on the therapeutic strategies used.

## 2. Materials and Methods

### 2.1. Animals

Wistar rats weighing between 350 and 450 g were used in this study. The animals were obtained from the animal facility of George Emil Palade UMFST of Targu Mures and were kept in standard laboratory conditions with a 12 h light/dark cycle, a temperature of 22 °C ± 2 °C, and a humidity of 60 ± 5%. The rats were housed in individual cages and provided standard pellet feed and water ad libitum. All animal procedures were performed according to the guidelines of the Institutional Animal Care and Use Committee, approved by the Ethical Committee of George Emil Palade UMFST of Targu Mures.

### 2.2. Three-Dimensional Nerve Guidance Conduits

A 3D nerve guidance conduit (NGC) was designed using computer-aided design (CAD) software (SolidWorks, Dassault Systèmes, France). The guide was printed using a fused deposition modeling (FDM) 3D printer (MakerBot Replicator, MakerBot Industries, New York, NY, USA) with a polylactic acid (PLA) filament. The guide had a length of 10 mm, a diameter of 1.5 mm, and 4 grooves on its surface to guide nerve regeneration. The guide was sterilized by autoclaving it prior to surgery.

### 2.3. Adipose Stem Cell Isolation and Culture

Adipose stem cells (ASCs) were isolated from the inguinal fat pads of the rats by the enzymatic digestion method, as previously described [22,23]. The cells were cultured in Dulbecco’s modified Eagle’s medium (DMEM, Gibco, Thermo Fisher Scientific, Waltham, MA, USA) supplemented with 10% fetal bovine serum (FBS, Gibco, Thermo Fisher Scientific, Waltham, MA, USA) and 1% penicillin/streptomycin (Gibco, Thermo Fisher Scientific, Waltham, MA, USA). The cells were incubated in a humidified atmosphere with 5% CO_2_ at 37 °C.

### 2.4. Cell Labeling

The ASCs were labeled with a fluorescent dye, DiI (1,1’-dioctadecyl-3,3,3’,3’-tetramethylindocarbocyanine perchlorate, Invitrogen, Thermo Fisher Scientific, Waltham, MA, USA), before transplantation. The cells were incubated with 5 μg/mL DiI in DMEM for 30 min at 37 °C. The labeled cells were washed with phosphate-buffered saline (PBS) and then trypsinized for transplantation.

### 2.5. Surgical Procedure

The surgeries for all the rats were performed under continuous general anesthesia with the following parameters: an induction chamber with a 0.8 L/min O_2_ flow and 5% isoflurane and, during surgery, inhalation anesthesia via a mask with a 0.6 L/min O_2_ flow and 2–3% isoflurane. The surgical procedure was performed under sterile conditions. The right sciatic nerve was exposed by making an incision in the skin overlying the biceps femoris muscle. The nerve was then transected at 10 mm from the sciatic notch. The proximal and distal stumps of the nerve were carefully trimmed to remove any epineurium and connective tissue, leaving only the fascicles of the nerve [24,25].

**Figure 1:**
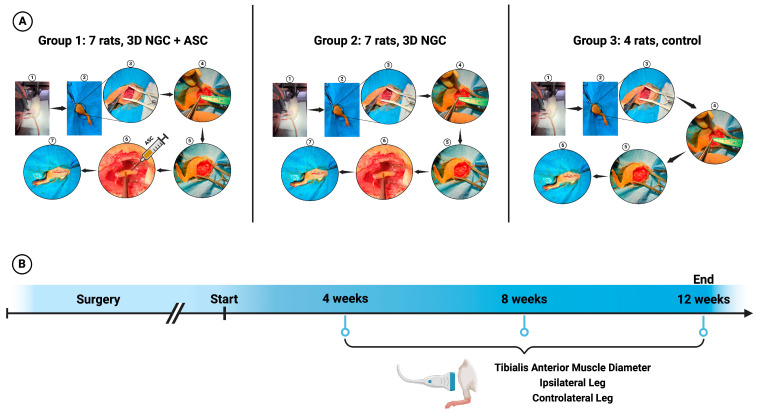
Graphic representation of the stages of this study: (**A**) a schematic presentation of the murine model used for each of the three groups and (**B**) a presentation of the method used to quantify muscle atrophy severity. To determine the severity of muscle atrophy, the diameter of the ipsilateral and contralateral anterior tibial muscles was quantified. The three groups share the same stages until a nerve defect of 10 mm is created at the sciatic nerve (stages 1–5). After that, a 3D NGC was used in the first two groups to bridge the gap between the two nerve ends, while the first group also received an injection of additional ASCs inside the 3D NGC.

The study group was divided into 3 subgroups (Figure 1):

- Group 1: 7 rats in which the 3D NGC was implanted into the gap between the proximal and distal stumps of the sciatic nerve. The guide was fixed in place with 10–0 nylon sutures. The ASCs (2 × 106 cells in 10 μL of PBS) were injected into the guide through one of the grooves.
- Group 2: 7 rats in which the 3D NGC was implanted into the gap between the proximal and distal stumps of the sciatic nerve. The guide was fixed in place with 10–0 nylon sutures without the use of ASCs.
- Group 3: 4 rats were used as a control group in which we only made a 10 mm defect at the level of the sciatic nerve without any reconstruction. We wanted to reduce the number of animals that were sacrificed unjustifiably. Additionally, based on published studies on PNI, four rats are often used for a control group [26,27,28].

Furthermore, the skin was then sutured using 4–0 nylon sutures.

### 2.6. Determination of Muscle Atrophy

We used ultrasound to measure the diameter of the tibialis anterior (TA) muscle in the lower limb with a nerve defect at 4 weeks, 8 weeks, and 12 weeks (Figure 1). The diameter of the contralateral limb was also measured for comparison. We determined the degree of atrophy by calculating the ratio between the diameter of the ipsilateral TA muscle and the diameter of the contralateral muscle.

### 2.7. Mechanical Testing

We collected TA muscle samples during animal sacrifice to analyze the biomechanical profile of muscle tissue distal to the nerve defect. These samples were preserved in PBS and analyzed within 6 h in our laboratory. The mechanical characteristics of the TA muscle were determined using the BioTester^®^ 5000 (CellScale, Waterloo, ON, Canada) at the Regenerative Medicine Laboratory of CCAMF within George Emil Palade UMFST of Targu Mures. The TA muscle was positioned along the longitudinal axis using a clamp mounting system with an initial separation of 5 mm. The examination protocol began with 10 cycles, which involved an elongation of 10% of the initial length at a rate of 1% per second, followed by a recovery phase. We then calculated the Cauchy stress and Young’s modulus based on data from the final cycle, using formulas available in the literature [29,30,31,32]. After the 10 cycles, the specimens were stretched until they failed to determine tensile strain and tensile stress at the point of rupture [29,30,31,32].

### 2.8. Statistical Analysis

For statistical analysis, we used IBM SPSS 28.0.1.0 for macOS (IBM software, Armonk, NY, USA). The Mann–Whitney U-test was used to calculate the difference in the mechanical characteristic parameters of the TA muscle between the groups at 10% stretch and failure. A *p*-value less than 0.05, which corresponds to a 95% confidence level, was considered significant. Additionally, the severity of muscle atrophy was compared between the groups using the Mann–Whitney U-test.

## 3. Results

**Figure 2:**
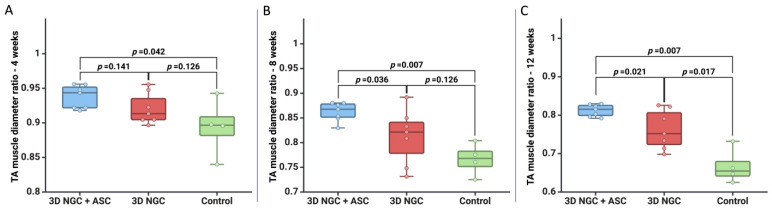
Graphic representation of muscle atrophy for each group at (**A**) 4 weeks, (**B**) 8 weeks, and (**C**) 12 weeks.

We conducted a study using a murine model to investigate peripheral nerve regeneration. We created a 10 mm sciatic nerve defect and divided the mice into three groups. In the first phase of the study, we evaluated the severity of muscle atrophy in each group. After four weeks, we observed more severe atrophy in the control group compared to the group that received treatment with 3D NGCs and ASCs (*p* = 0.042). At eight weeks, the control group and the 3D NGC group showed more severe muscle atrophy (*p* = 0.007 and *p* = 0.036, respectively) compared to the group that received the 3D NGC + ASC treatment. Moreover, at 12 weeks, the control group showed the most severe atrophy, followed by the 3D NGC group, while the group treated with ASCs showed the smallest deficit (Figure 2).

**Figure 3:**
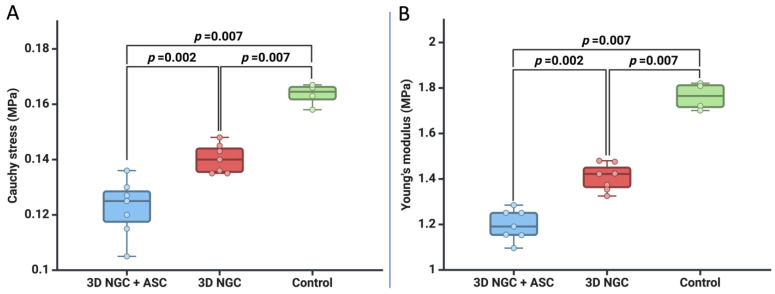
The mechanical characteristics of the TA muscle at a 10% stretch: (**A**) Cauchy stress (MPa) and (**B**) Young’s modulus (MPa).

Following that, after 12 weeks, we compared the mechanical characteristics of the TA muscle obtained from the limb with the nerve defect between the three groups. At 10% stretch, we found that the control group had the highest Cauchy stress values compared to both the 3D NGC group (0.164 MPa vs. 0.141 MPa, *p* = 0.007) and the 3D NGC + ASC group (0.164 MPa vs. 0.123 MPa, *p* = 0.007). Moreover, the 3D NGC group had a higher average value of Cauchy stress than the group in which we used ASCs (0.141 MPa vs. 0.123 MPa, *p* = 0.002). In addition, we found that the control group had the highest TA muscle stiffness, followed by the 3D NGC group, and the best muscle elasticity was in the group in which we used ASCs. The mean Young’s modulus for the control group was 1.763 MPa, while for the 3D NGC group, it was 1.402 MPa (*p* < 0.001), and it was 1.147 MPa for the 3D NGC + ASC group (*p* = 0.007), as shown in Figure 3B.

**Figure 4:**
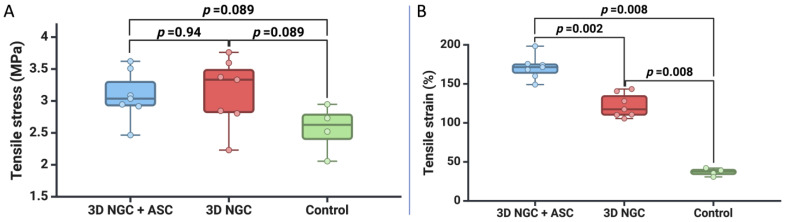
The mechanical characteristics of the TA muscle at a failure: (**A**) tensile stress (MPa) and (**B**) tensile strain (%).

Moreover, we analyzed the biomechanical behavior of the TA muscle until failure and found that for tensile stress, the control group had lower values compared to the other two groups, but the difference was not statistically significant (*p* = 0.089 for both). However, in terms of tensile strain, we observed greater compliance in the 3D NGC + ASC group compared to the other two groups (*p* = 0.002 and *p* = 0.008). Additionally, we found greater compliance in the 3D NGC group compared to the control group (*p* = 0.008) (Figure 4).

**Figure 5:**
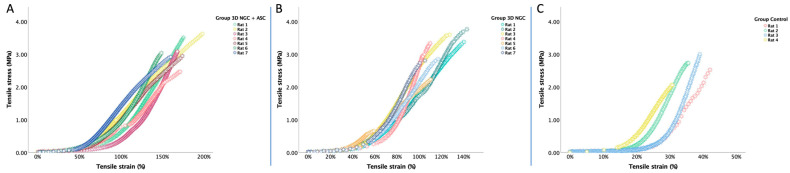
Mechanical behavior at failure obtained from uniaxial analysis of all TA muscle specimens from the (**A**) 3D NGC + ASC, (**B**): 3D NGC, and (**C**) control groups.

In Figure 5, a stress–stretch plot for each TA muscle from the three groups is presented to highlight the mechanical behavior. As shown in Figure 5A, the TA muscle samples from the 3D NGC + ASC group demonstrate better compliance and a higher degree of elasticity compared to the other two groups (*p* = 0.002 and *p* = 0.008). Furthermore, in the 3D NGC batch (Figure 5B), though we recorded tensile stress similar to the first group (3.135 MPa vs. 3.082 MPa, *p* = 0.94), muscle stiffness is observed, and the average value of tensile strain is 122.22%. Lastly, in the control group (Figure 5C), we recorded the most significant stiffness, with a mean value of tensile strain of 36.85%.

## 4. Discussion

The main result of this study is the demonstration for the first time, according to our knowledge, that the use of 3D NGCs and ASCs in PNI not only promotes nerve regeneration but also has a significant impact on the compliance of the distal musculature at a 10% stretch as well as at stretching to failure. Furthermore, the therapeutic strategy we proposed limits muscle atrophy, which can be beneficial for the post-operative recovery process.

Following PNI, there exists a certain degree of inherent capacity for repair and regeneration, but recovering from severe nerve injuries can still be difficult, and the results achieved thus far have not been entirely satisfactory [33,34,35]. Additionally, for important nerve defects, a nerve autograft is the gold standard, but it has limitations such as donor site morbidity, a limited supply of donor nerves, and a lack of size congruence [36,37].

Special interest has been given to the identification and development of nerve guide conduits (NGCs) that can guide the nerve fibers and accelerate the regeneration process. The most common approaches used in developing NGCs are freeze-drying [38], electrospinning [39], and 3D printing [6,7,8,9], but the results have been mixed, and an optimal NGC for surgical practice has not yet been developed. However, the use of stem cells in the NGC has shown promise in accelerating the regeneration process [9,40]. In recent decades, stem cells have been used to fabricate complex structures such as vascular topologies, cardiac patches, and even hearts, indicating their potential applications in tissue engineering [41,42,43,44,45,46]. Thus, Rhode et al. [47] reported the effects of different types of nerve guide conduits with adipose stem cells on peripheral nerve regeneration in a meta-analysis of 17 studies between 2016 and 2020. The authors showed that adipose stem cells promote axonal regeneration, myelin formation, and the restoration of the denervated muscle in most of the studies.

Delaying nerve reconstruction prolongs the period of muscle tissue denervation and worsens the degree of muscle atrophy [48]. Li et al. [49] demonstrated that mean tensions in the muscle contraction force test in a group of animals with immediate nerve repair were higher than a group with delayed repair (*p* < 0.05). Furthermore, Hao et al. [50] induced muscle atrophy via the local injection of botulinum toxin-A in a rat femur fracture model. The authors discovered that the presence of muscle atrophy has a negative impact on the healing of fractures as well as on the biomechanical characteristics of the bone. Additionally, studies have shown that muscle atrophy is associated with prolonged mechanical ventilation in severe trauma patients [20] and with the decline in bone health in the elderly [51]. Regarding the mechanical characteristics of muscle tissue, they play an important role in post-operative recovery. Eken et al. [52] demonstrated that muscle atrophy correlates with poor functional outcomes following Achilles tendon repair.

It is important to note that our study has several limitations that must be considered. Firstly, we did not conduct a histological analysis of the muscle samples due to the biomechanical analysis of the TA muscle until failure. Additionally, we only used one concentration of ACSs (2 × 10^6^). In the future, we plan to test higher concentrations to determine if there is a greater benefit. We aim to further develop this preliminary study and introduce the structural remodeling of the muscle tissue, as well as study the evolution of the muscles and the mechanical behavior of the contralateral TA muscle. Furthermore, we propose to administer injectable ACSs at the level of the distal muscles and monitor the structural and biomechanical remodeling immediately, post-operatively, and at well-defined intervals.

## 5. Conclusions

Our study demonstrates that the combination of 3D NGCs and ASCs increases the process of nerve regeneration and significantly improves the compliance and mechanical characteristics of muscle tissue distal to the injury site in a PNI murine model. Moreover, this therapeutic approach helps to prevent muscle atrophy, which may ultimately facilitate the recovery of limb function.

## Data Availability

The data that support the findings of this study are available from the corresponding author upon reasonable request.
